# ICTV Virus Taxonomy Profile: *Tristromaviridae*

**DOI:** 10.1099/jgv.0.001190

**Published:** 2018-12-12

**Authors:** David Prangishvili, Elena Rensen, Tomohiro Mochizuki, Mart Krupovic, ICTV Report Consortium

**Affiliations:** ^1^​ Department of Microbiology, Institut Pasteur, 25 Rue du Dr. Roux, 75015 Paris, France; ^2^​ Department of Cell Biology and Infection, Institut Pasteur, 25 Rue du Dr. Roux, 75015 Paris, France; ^3^​ Earth-Life Science Institute, Tokyo Institute of Technology, Tokyo, 152-8550, Japan

**Keywords:** *Tristromaviridae*, ICTV Report, taxonomy

## Abstract

*Tristromaviridae* is a family of viruses with linear, double-stranded DNA genomes of 16–18 kbp. The flexible, filamentous virions (400±20 nm×30±3 nm) consist of an envelope and an inner core constructed from two structural units: a rod-shaped helical nucleocapsid and a nucleocapsid-encompassing matrix protein layer. Tristromaviruses are lytic and infect hyperthermophilic archaea of the order Thermoproteales . This is a summary of the International Committee on Taxonomy of Viruses (ICTV) Report on the *Tristromaviridae*, which is available at www.ictv.global/report/tristromaviridae.

## Abbreviation

VP, Virion protein.

## Virion

**Fig. 1.**
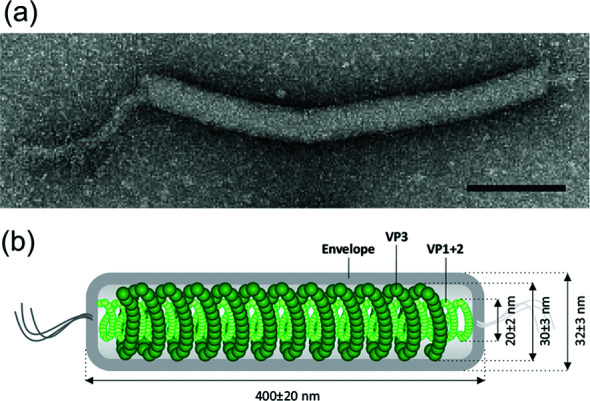
(a) Electron micrograph of a virion of Pyrobaculum filamentous virus 1 negatively-stained with 2 % uranyl acetate. (b) Schematic of the virion organization with the position of the three major capsid proteins indicated. Modified with permission from [1].

The virions are filamentous, 400±20×32±3 nm (Table 1, Fig. 1), and contain a lipid envelope and an inner core consisting of two structural units: (i) a rod-shaped helical nucleocapsid, formed of two major virion proteins (VP1 and VP2), each with a molecular mass of 14 kDa, and (ii) a nucleocapsid-encompassing protein sheath composed of a single virion protein (VP3) of 18 kDa [1]. The sheath layer is sandwiched between the nucleocapsid and the lipid envelope, akin to the matrix protein layer found in some eukaryotic, negative-sense RNA viruses. The virions also contain at least five minor proteins with molecular masses in the range of 11–30 kDa.

**Table 1.** Characteristics of the family *Tristromaviridae*

Typical member: Pyrobaculum filamentous virus 1 (KU307456), species * Pyrobaculum filamentous virus 1*, genus *Alphatristromavirus*	
Virion	Filamentous, flexible (400±20 nm×30±3 nm) particle consisting of a nucleoprotein core covered by a matrix protein layer and enveloped with a lipid membrane; bundles of thin filaments are attached to both ends
Genome	Linear, double-stranded DNA genomes of 16–18 kbp
Replication	Viruses are lytic; virions are released by the rupture of the host cell envelope
Translation	Not characterized
Host range	Hyperthermophilic archaea of the order Thermoproteales
Taxonomy	One genus, two species

## Genome

**Fig. 2.**
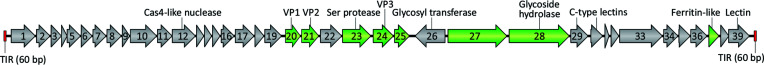
Genome map of Pyrobaculum filamentous virus 1 (17 714 bp). Arrows indicate ORFs and the direction of transcription. Genes encoding structural proteins are shown in green. Terminal inverted repeats (TIR) are depicted by red rectangles. Modified with permission from [1].

The linear dsDNA genome of Pyrobaculum filamentous virus 1 is 17 714 bp, including 60 bp terminal inverted repeats, and is predicted to encode 39 proteins (Fig. 2), most of which do not show similarities to the sequences in public databases [2]. Nine gene products of Pyrobaculum filamentous virus 1 share significant sequence similarity with proteins encoded in the partially sequenced genome of Thermoproteus tenax virus 1 [3, 4].

## Replication

Virions bind to the host cell via the interaction of terminal protrusions with the host pili-like appendages. The viral genome is present in the host cells in a linear non-integrated form and mature virions assemble in the host cell lumen prior to release. The virus is lytic and virions are released by the rupture of the host cell envelope. The mechanism of genome replication remains unknown [1].

## Taxonomy

The family *Tristromaviridae* comprises a single genus, *Alphatristromavirus*, with two species. Tristromaviruses infect members of the hyperthermophilic archaeal order Thermoproteales . Thermoproteus tenax virus 1 [5] infects members of the genus *Thermoproteus ,* and Pyrobaculum filamentous virus 1 infects members of the genus *Pyrobaculum *. Thermoproteus tenax virus 1 was formerly classified in the family *Lipothrixviridae* [6].

## Resources

Full ICTV Report on the family *Tristromaviridae*: www.ictv.global/report/tristromaviridae.
